# Modelling maintenance scheduling strategies for highway networks

**DOI:** 10.1371/journal.pone.0269656

**Published:** 2022-06-08

**Authors:** Bao Tong, Jianwei Wang, Xue Wang, Feihao Zhou, Xinhua Mao, Yaxin Duan

**Affiliations:** 1 College of Transportation Engineering, Chang’an University, Xi’an, China; 2 Engineering Research Center of Highway Infrastructure Digitalization, Ministry of Education, Xi’an, China; 3 Engineering Research Center of Digital Construction and Management for Transportation Infrastructure of Shaanxi Province, Xi’an, China; 4 Xi’an Key Laboratory of Digitalization of Transportation Infrastructure Construction and Management, Xi’an, China; National Taiwan University of Science and Technology, TAIWAN

## Abstract

Although a wide range of literature has investigated the network-level highway maintenance plans and policies, few of them focused on the maintenance scheduling problem. This study proposes a methodology framework to model and compare two different maintenance scheduling strategies for highway networks, i.e., minimal makespan strategy (MMS) and minimal increased travel delay strategy (MITDS). We formulate MMS as a mixed integer linear programming model subject to the constraints of the quantity of manpower and the worst-first maintenance sequence. A bi-level programming model is proposed to quantify and optimize MITDS. The upper level model determines the optimal scheduling to minimize the increased traffic delays during the maintenance makespan. In the lower level, a modified day-to-day traffic assignment model is put forward to reflect the traffic evolution dynamics by simulating travelers’ route choice behaviors. A simulated annealing algorithm and augmented Lagrange algorithm are employed to solve the two proposed models, respectively. Finally, a numerical example using a highway network is developed. The two proposed strategies are tested considering different traffic demands, numbers of engineering teams, and travelers’ sensitivities to traffic congestion. The experiment results reveal that compared with MMS, MITDS extends makespan by 2 days though, it reduces the total increased travel delays by 4% and both MMS and MITDS can obtain the minimum total increased travel delays when the number of engineering teams is 6. The sensitivity analysis indicates that both the two strategies have the maximum and minimum total increased travel delays when the weight of prediction in travelers’ perception is 0.3 and 0.7, respectively. The proposed framework has the potential to provide reference in implementing highway maintenance activities reasonably.

## Introduction

Highway is a kind of indispensable infrastructure that plays an important role in boosting the economic development and increasing mobility. Routine maintenance is necessary to guarantee this infrastructure system at a high service level. However, due to the large scale of highway assets and rapid condition deterioration, highway maintenance management has become a challenge to the transportation agencies [1]. In view of this, numerous studies in recent years have been conducted focusing on network-level maintenance optimization [2–4]. To our best knowledge, most approaches in the literature define the highway maintenance management as a problem of budget constrained maintenance strategy optimization [5], which can generate plans, e.g., types of maintenance actions [6], budget allocation scheme [7], maintenance sequence [8], and intensity of the maintenance actions [9], etc. over a planning horizon. Such maintenance plans are optimal, but cannot provide effective reference in the practice of highway maintenance implementation because they are not involved with a detailed maintenance activity scheduling in a very time period, i.e., they cannot identify the makespan of the entire maintenance project (a maintenance project includes a set of maintenance activities), the start time of each maintenance activity or the number of engineering teams. This constitutes the main motivation of the study.

**Fig 1:**
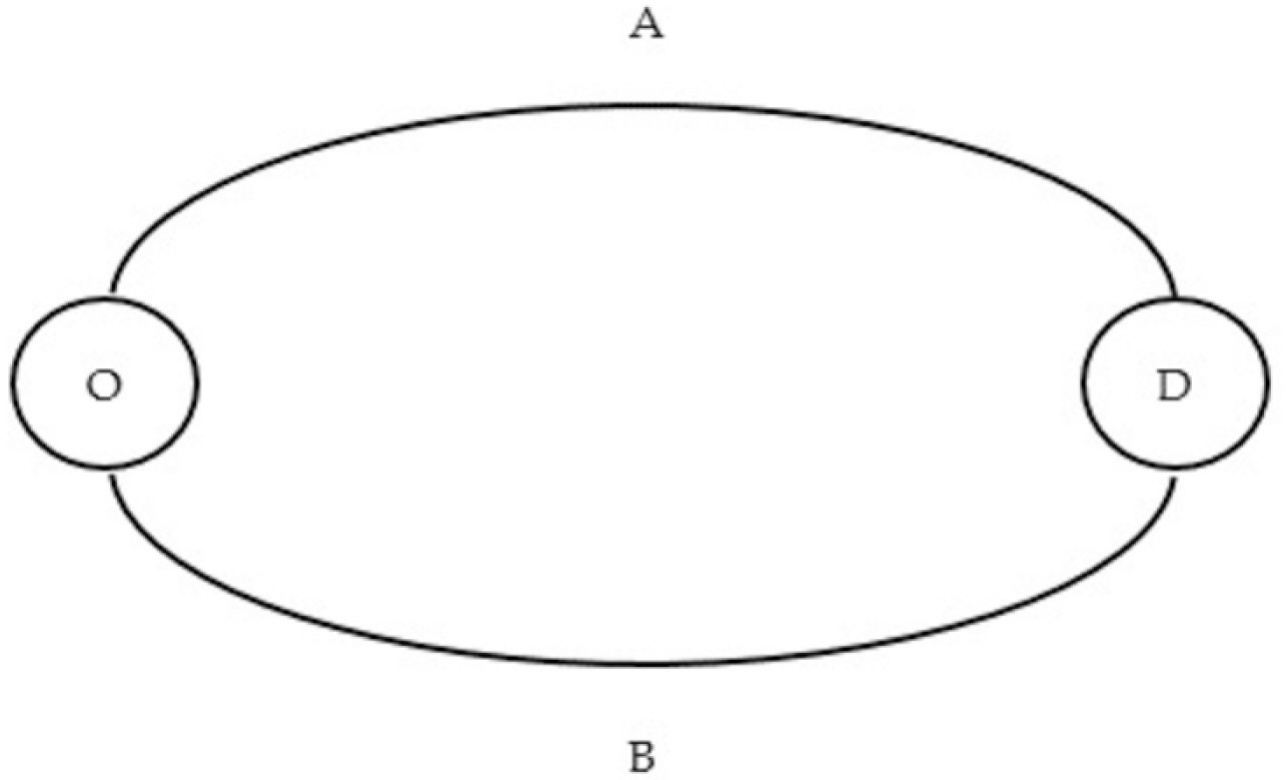
Hypothetical traffic network.

The highway network maintenance scheduling (HNMS) problem based on the given optimal maintenance plans is concerned with scheduling a set of maintenance activities in order to achieve a certain objective (or multiple objectives) under the constraints of limited resources, precedence relations of maintenance activities, etc., which makes it as a resource-constrained project scheduling problem (RCPSP). Most of the existing studies on the RCPSP deal with the minimization of the makespan as the objective [10]. However, in the case of HNMS, it is possible to increase the travel delays when minimal maintenance makespan is realized because of the dynamic traffic flow distribution caused by lane closure during the maintenance period. To further explain the possibility, a hypothetical highway network consisting of two parallel links plotted in Fig 1 is developed as a simple example. Assume that link A and link B have homogeneous physical characteristics and conditions. The daily traffic demand between O and D is *N*, which remains constant during the maintenance period. The equilibrium traffic flows on link A and link B are both *q*_0_ per day, 2*q*_0_ = *N*, and the average travel time on the two links are both *t*_0_ in the pre-maintenance period. Maintenance activities on link A and link B bring about the same amount of traffic capacity loss and their maintenance durations are both *n* days.

**Fig 2:**
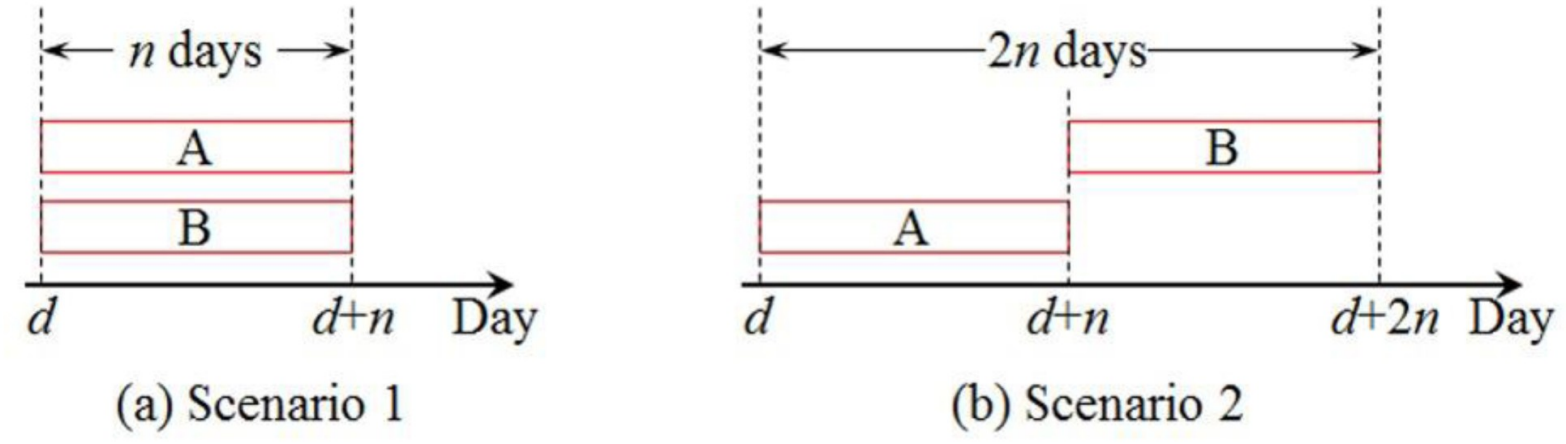
Two scenarios of highway maintenance scheduling.

We consider the following two scenarios of highway maintenance scheduling shown in Fig 2.

- Scenario 1: link A and link B are maintained simultaneously. The equilibrium traffic flows on link A and B during the maintenance period are both *q*_1_ per day, 2*q*_1_ = *N*, and the average travel time on the two links are both *t*_1_, *t*_1_> *t*_0_.
- Scenario 2: link B is maintained after link A with zero time-lags. When link A is maintained, the equilibrium traffic flows on link A and link B are *q*_2_ and *q*_3_ per day, respectively, *q*_2_<*q*_3_, *q*_2_+*q*_3_ = 2*q*_1_ = *N*, and the average travel time on the two links are *t*_2_ and *t*_3_, respectively, *t*_2_>*t*_3_. The same is true when link B is maintained.

It is not difficult to find that in Scenario 1, the maintenance makespan is *n* days, and the sum of the increased traffic delays in the makespan is 2*n*(*q*_1_*t*_1_-*q*_0_*t*_0_). While in Scenario 2, the maintenance makespan is 2*n* days, and the sum of the increased traffic delays in the makespan is 2*n*(*q*_2_*t*_2_+*q*_3_*t*_3_-2*q*_0_*t*_0_). Apparently, when *q*_2_*t*_2_+*q*_3_*t*_3_<*q*_1_*t*_1_+*q*_0_*t*_0_, Scenario 1 has minimal makespan but produces more increased traffic delays, which reveals that a HNMS aiming to minimize the makespan could lead to more traffic congestion and traffic delays. Actually, this problem in the real world is even more complicated. Hence, a method framework for modeling the optimal HNMS scheduling strategy is highly desired to extend the methodology of highway maintenance management. Unfortunately, such an approach was rarely investigated in the existing literature to our best knowledge. The detailed analysis of the previous literature can be seen in Section 2.

From the analysis results of the aforementioned example, we know that minimum makespan cannot ensure the minimization of total increased travel time caused by the maintenance activities. It is necessary to examine in what maintenance scenarios the objectives of minimal makespan and minimal increased travel delay are appropriate. To this end, this study proposes a mathematical programming framework to model and compare two different maintenance scheduling strategies for highway networks, i.e., minimal makespan strategy (MMS) and minimal increased travel delay strategy (MITDS). The work makes the following three contributions. (1) We employ mixed integer linear programming to formulate MMS, and propose a bi-level model, which consists of a mixed integer nonlinear programming model in the upper level to minimize the increased traffic delays during the makespan and a day-to-day traffic assignment model at the lower level to simulate the traffic evolution when maintenance activities begin and end. (2) A modified simulated annealing algorithm and an augmented Lagrange algorithm are designed to solve MMS model and MITDS model, respectively. (3) A numerical example using a highway network is utilized to help understanding the performance of the two strategies, and provides potential aid for transportation agencies to make the optimal maintenance scheduling schemes.

The remainder of this paper is organized as follows: Section 2 reviews the studies on RCPSP. Section 3 presents the models of MMS and MITDS. The model solution algorithms are provided in Section 4. In Section 5, we develop a numerical example to compare the features of the two models. Conclusions and future work are discussed in Section 6.

## Literature review

Resource-constrained project scheduling problem (RCPSP) is a classic problem in project management, which aims to identify the start and completion times of a set of sequential activities by the allocation of limited resources [11]. Since Pritsker et al. [12] firstly introduced a zero-one programming model to deal with the multi-project scheduling, a variety of studies in this realm have been investigated. RCPSP can be classified into different variants by the number of projects and/or execution modes (multiple or single), the type of resources (renewable, unrenewable or doubly resource constrained), the precedence relationship among activities (finish-to-start, finish-to-finish, start-to-start or start-to-finish), the characteristics of scheduling (preemptive or non-preemptive), whether the duration of each activity is deterministic or stochastic, and whether the processing time and/or resource consumption is discrete or continuous [10]. To our best knowledge, most of the existing studies of RCPSP focus on the doubly resource constrained single project scheduling with finish-to-start precedence relationship [1, 2, 5, 13–17]. As reported in literature, the RCPSP can be modeled using two methods, i.e., activity-based method and event-based method [18]. The former establishes models using the variables indexed by time based on a precedence activity-on-node network with *n* real activities and 2 dummy activities having zero duration and resource consumption [19]. The latter proposes models by variables indexed by events, wherein events correspond to start or end times of activities, hence the number of events is *n*+1 [20]. Additionally, event-based method does not have to use the dummy activities. The traditional RCPSP is formulated as a model with the minimization of makspan subject to three main constraints, i.e., the precedence relationship constraint, resource availability constraint and temporal constraint [21]. Aside from minimal makespan, some other objectives are considered as well, e.g., minimization of total weighted tardiness [22], maximal net present value [23], minimal resources availability costs [24], etc. Additionally, some multi-objective models are also employed, e.g., Gomes et al. [25] established a bi-objective model with minimal makespan and minimization of the total weighted start time of the activities. As noted by Thomas and Salhi [26], the RCPSP is a NP-hard combinatorial optimization problem. The exact algorithms such as linear programming and branch-and-bound technique are feasible to solve this problem, but only effective for those small-sized problems with less than 60 activities [27]. For large-sized instances, it is proved that heuristic algorithms and more optimal solutions have better performance [28].

Highway maintenance activities usually involve in the lane closure reducing highway capacity and decreasing speed limit, which aggravates the congestion and increases the user costs. To mitigate the negative effect, some of the above optimization methods have been applied into the HNMS. Relevant studies are summarized in Table 1. However, there is a small body of literature focusing on HNMS in the past two decades. It is found that two main types of methods are utilized, i.e., optimization method and simulation-based method. Specifically, Cheu et al. [29] developed a zero-one programming model with minimization of the total travel time solved by a hybrid genetic algorithm, and Lee [30] applied the ant colony algorithm to search for a near-optimal schedule with minimal traffic delay simulated by microscopic simulator VISSIM. Despite their contributions, two limitations still exist. (1) Metaheuristic methods, especially genetic algorithms, are the most commonly used to solve the optimization models of HNMS. However, metaheuristic methods cannot ensure the global optimality of the solution [31]. (2) Most of the studies listed in Table 1 assume that the traffic flow distribution in the highway network experiences a significant change of equilibrium state from pre-maintenance period to post-maintenance period. The only exception is Yang et al. [32], where a gradual transition of traffic flow distribution was taken into account, but the proposed model failed to deal with the maintenance activity precedence relationship constraint, makespan constraint or manpower constraint.

**Table 1:** Summarization of the existing studies of HNMS.

Study	Model	Solution Approach
Fwa et al. [13]	A zero-one programming model with minimization of the total traffic delay subjective to activity duration constraint, makespan constraint and manpower constraint.	genetic algorithm
Ahire et al. [33]	A mixed integer programming model with minimization of the makespan subject to workforce availability constraint and maintenance time window constraint.	evolution strategies
Chang et al. [14]	A linear programming model with minimization of the total traffic delay simulated by dynamic traffic assignment approach subjective to manpower constraint.	tabu search method
Chien et al. [34]	Uses a method integrating numerical and analytical approaches, where a total cost objective function is formulated and a tabu search algorithm is developed to search the best starting time of each maintenance project	
Wang et al. [15]	Uses a hybrid genetic algorithm to generate and select the optimal maintenance schedule with minimal increased travel time, and utilizes a microscopic simulator PARAMICS to estimate the traffic delay of each schedule.	
Cheu et al. [29]	A zero-one programming model with minimization of the total travel time subjective to activity duration constraint, makespan constraint and manpower constraint.	hybrid genetic algorithm
Tang and Chien [16]	A linear programming model with minimization of total cost subjective to maintenance time window constraint, makespan constraint and highway length constraint.	genetic algorithm
Lee [30]	Calculates the traffic delay of vehicles by microscopic simulator VISSIM, and applies team ant colony optimization to search for a near-optimal schedule with minimal traffic delay.	
Chien and Tang [17]	A mix-integer model with minimization of the sum of agency cost and user cost subject to activity duration constraint, highway length constraint and makespan constraint.	genetic algorithm
Gong and Fan [35]	A bi-level model. The upper level model minimizes the total travel delay subject to maximum project deadline and project starting time constraint. The low level model is a deterministic user equilibrium model.	genetic algorithm
Yang et al. [32]	A non-linear programming model with minimization of total increased travel cost subject to traffic demand constraint and traffic flow conservation constraint.	genetic algorithm

In reality, traffic flows have an evolution process in case of a perturbation [36]. When a set of pavement maintenance activities with long durations are started and completed, the highway network has day-to-day traffic dynamics, especially during the transitory periods, i.e., from pre-maintenance period to post-maintenance period and from post-maintenance period to recovery period.

## Formulation of the problem

### Problem statement

The problem considers the maintenance scheduling optimization of a highway network based on the given maintenance plans, that is, types of maintenance actions, cost of each maintenance activity, budget allocation scheme, maintenance sequence, number and location of each link to be maintained are known. All links in the network are assumed to have deterministic but different physical characteristics and conditions i.e., length, free-flow travel time, free-flow speed, traffic capacity. For simplicity, we also assume that the daily traffic demands in the network is constant that does not vary over time. In the pre-maintenance period, the traffic distribution in the network reaches a user equilibrium (UE) state where traffic flows and vehicles’ average travel time can be calculated.

When maintenance actions begin, traffic capacities on the maintenance links drop immediately to a lower level due to the lane closure, which brings the network to a new condition. Responding to the perturbation in the network conditions, travelers reselect the optimal paths depending on their previous experience and the prediction of traffic delays [37], which makes the network traffic gradually evolve into a new UE state [38]. This also happens at the end of maintenance actions. The duration of each maintenance activity is a known parameter. Every single maintenance activity is nonpreemptive and start only once during the entire maintenance period. Since worst-first (WF) is the most widely used highway maintenance strategy [39], we adopt the WF maintenance strategy in this study, which makes the precedence relationship among the maintenance activities as start-to-start (SS) for all the maintenance activities and finish-to-start (FS) for the maintenance activities assigned to the same engineering team. We define the coping abilities of engineering teams as renewable resources, which cannot be shared among the maintenance activities simultaneously, i.e., every single engineering team cannot deal with more than one maintenance activity at the same time.

### Notation list

The notations used in this study are given as follows:

**Sets and indices**
*A*  Set of all links in the highway network, indexed by *a*∈*A*.
*I*  Set of links to be maintained, indexed by *i*,*j*∈*I*.
*S*  Set of engineering teams, indexed by *s*∈*S*.
*P*  Set of SS precedence relationship of maintenance activities.
*P*_*s*_  Set of FS precedence relationship of maintenance activities assigned to engineering team *s*, *P*_*s*_⊆*P*.
*W*  Set of origin-destination (OD) pairs, indexed by *w*∈*W*.
*K*_*w*_  Set of paths that connect OD pair *w*, indexed by *k* = *K*_*w*_.

**Parameters**
*t*  Time period, *t* = 1,2,⋯,*T*.
*T*  Length of a discrete time period.
*q*_*w*_  Traffic demand of OD pair *w*.
*d*_*i*_  Duration of maintenance activity *i*, i.e., maintenance activity for link *i*.
$c_{a}^{t}$  Traffic capacity of link *a* at time *t*.
$c_{a}^{0}$  Pre-maintenance traffic capacity of link *a*.
*ρ*_*i*_  Impact of maintenance activity on traffic capacity of link *i*, 0<*ρ*_*i*_<1.
$Z_{a}^{0}$  Pre-maintenance travel time of link *a* under the UE state.
$Z_{a}^{t}$  Travel time of link *a* at time *t*.
$z_{a}^{t}$  Free-flow travel time of link *a* at time *t*.
$z_{a}^{0}$  Pre-maintenance free-flow travel time of link *a*.
*σ*_*i*_  Impact of maintenance activity on free-flow travel time of link *i*, 0<*σ*_*i*_<1.
*α*, *β*  Traffic delay coefficients of BRP function.
*ξ*  Step-size parameter representing the weight of prediction in travelers’ perception, 0<*ξ*<1.
$q_{a}^{t*}$  Positive constant parameter representing the target traffic flow on link *a* at time *t*.

**Decision variables**
*x*_*ist*_  Binary variable that means if maintenance activity *i* assigned to engineering team *s* starts at time *t*, *x*_*ist*_ = 1; otherwise, *x*_*ist*_ = 0.
*M*  Makespan of the maintenance plan.
*Z*  Total increased travel delays during the maintenance makespan.
*Z*_*t*_  Total increased travel delays at time *t*.
$q_{a}^{t}$  Traffic flow on link *a* at time *t*.
$h_{kw}^{t}$  Traffic flow on the path *k* that connects OD pair *w* at time *t*.
*δ*_*akw*_  Binary variable that means if link *a* lies on path *k* that connects OD pair *w*, *δ*_*akw*_ = 1; otherwise, *δ*_*akw*_ = 0.

### Formulation of MMS

Next, the MMS is formulated as a mixed linear programming model to generate an optimal maintenance scheduling scheme of highway networks minimizing the makespan.


$$\text{min}M={\text{max}}_{i\in I,s\in S}\;\left\{\sum_{t=1}^{T}t∙x_{ist}+d_{i}\right\}$$ (1)

$$s.t.\;\sum_{s\in S}\sum_{t=1}^{T}t∙x_{ist}\leq \sum_{s\in S}\sum_{t=1}^{T}t∙x_{jst},\forall \left(i,j\right)\in P$$ (2)

$$\sum_{t=1}^{T}t∙x_{ist}+d_{i}\leq \sum_{t=1}^{T}t∙x_{jst},\forall \left(i,j\right)\in P_{s},\forall s\in S$$ (3)

$$\sum_{t=1}^{T}t∙x_{ist}+d_{i}\leq T,\forall i\in I,\forall s\in S$$ (4)

$$\sum_{t=1}^{T}t∙x_{ist}=1,\forall i\in I,\forall s\in S$$ (5)

$$0\leq \sum_{i\in I}\sum_{\tau =\text{max}\left\{1,t-d_{i}+1\right\}}^{t}x_{ist}\leq 1,\;\forall s\in S,\forall t\in T$$ (6)

$$0\leq \sum_{i\in I}\sum_{s\in S}\sum_{\tau =\text{max}\left\{1,t-d_{i}+1\right\}}^{t}x_{ist}\leq \left|S\right|,\;\forall t\in T$$ (7)

$$0\leq \sum_{i\in I}\sum_{s\in S}x_{ist}\leq \left|I\right|-\sum_{i\in I}\sum_{s\in S}\sum_{\tau =\text{max}\left\{1,t-d_{i}+1\right\}}^{t}x_{ist}-\sum_{i\in I}\sum_{s\in S}\sum_{\tau =0}^{\text{max}\left\{0,t-d_{i}+1\right\}}x_{ist},\forall t\in T$$ (8)

$$x_{ist}\in \left\{0,1\right\},\;\forall i\in I,\forall s\in S,\forall t\in T$$ (9)

In the MMS model, each maintenance activity starts at the time $\sum_{t=1}^{T}t∙x_{ist}$ and has to be finished at the time $\sum_{t=1}^{T}t∙x_{ist}+d_{i}$ with a deterministic duration *d*_*i*_. Among the end time of all highway maintenance activities, the maximum end time ${\text{max}}_{i\in I,s\in S}\;\left\{\sum_{t=1}^{T}t∙x_{ist}+d_{i}\right\}$ is defined as the maintenance makespan *M*. The objective function (1) aims to minimize the maintenance makespan. (*i*,*j*)∈*P* defines the precedence relation between the maintenance activities *i* and *j* indicating that *j* cannot be started before *i* is started. Eq (2) ensures the SS precedence relation between any two maintenance activities. Similarly, Eq (3) represents the SS precedence relation between any two maintenance activities assigned to engineering team *s*. Both Eqs (2) and (3) guarantee the WF maintenance strategy adopted in this study. Eq (4) ensures that each maintenance activity must be finished before the end of the given time period, i.e., by time *T*. Non-preemptive constraint (5) indicates that each maintenance activity must be implemented only once without interruption. $\sum_{i\in I}\sum_{\tau =\text{max}\left\{1,t-d_{i}+1\right\}}^{t}x_{ist}$ in constraints (6) is denoted as the ongoing maintenance activities at time *t*. These constraints ensure that each engineering team can carry out at most one maintenance activity at a time. Because of the limited availability of manpower, Eq (7) guarantees that the amount of the simultaneous maintenance activities cannot exceed the number of engineering teams at time *t*. $\sum_{i\in I}\sum_{s\in S}\sum_{\tau =0}^{\text{max}\left\{0,t-d_{i}+1\right\}}x_{ist}$ in Eq (8) defines the maintenance activities that have been finished by time *t* so that $\left|I\right|-\sum_{i\in I}\sum_{s\in S}\sum_{\tau =\text{max}\left\{1,t-d_{i}+1\right\}}^{t}x_{ist}-\sum_{i\in I}\sum_{s\in S}\sum_{\tau =0}^{\text{max}\left\{0,t-d_{i}+1\right\}}x_{ist}$ limits the upper bound of the number of maintenance activities that can be chosen to start. Finally, Eq (9) defines the types of decision variables.

### Formulation of MITDS

In this subsection, we propose a bi-level model taking the dynamic traffic flow distribution into consideration to formulate the MITDS. Using the information from the lower level model about the travelers’ travel time that can be estimated considering the upper level decisions [38], the upper level model determines the optimal highway maintenance activity scheduling to minimize the increased travel delays during the makespan. In the lower level model, travelers make the optimal path choices following the upper level decision as well as their previous experience and prediction of traffic delays.

#### Upper level model

$$\text{min}Z=\sum_{t=1}^{M}(Z_{t}-Z_{t}^{*})$$ (10)

s.t.

Eqs (2)–(9).

The upper level is a mixed nonlinear programming model. The objective function Eq (10) minimizes the increased travel delays during the makespan *M*. We define $Z_{t}-Z_{t}^{*}$ as the increased travel delays at the time *t*. *Z*_*t*_ is the total travel time in the network at the time *t*, which is obtained from the lower level model. $Z_{t}^{*}$ is total travel time at the time *t* if no maintenance activity is implemented in the network. Since it is assumed that the traffic demands is a constant parameter, any $Z_{t}^{*}(\forall t=1,2,\cdots ,M)$ equals to the daily travel time $Z_{0}^{*}$ in the pre-maintenance period under the UE state. Hence, objective function (10) can be replaced by Eq (11).


$$\text{min}Z=\sum_{t=1}^{M}Z_{t}-MZ_{t}^{*}$$ (11)

#### Lower level model


$$\text{min}Z_{t}=\sum_{a\in A}\int_{0}^{q_{a}^{t}}Z_{a}^{t}(v,c_{a}^{t})dv,\forall t\in T$$ (12)

$$s.t.\sum_{k\in K_{w}}h_{kw}^{t}=q_{w},\forall w\in W,\forall t\in T$$ (13)

$$\sum_{w\in W}\sum_{k\in K_{w}}h_{kw}^{t}∙\delta_{akw}^{t}=q_{a}^{t},\forall a\in A,\forall t\in T$$ (14)

$$q_{a}^{t+1}=q_{a}^{t}+\xi \left(q_{a}^{t+1*}-q_{a}^{t}\right),\forall a\in A,\forall t\in T$$ (15)

$$h_{kw}^{t}\geq 0,\forall w\in W,k\in K_{w},\forall t\in T$$ (16)

$$c_{a}^{t}=c_{a}^{0},\forall a\in (A-I),\forall t\in T$$ (17)

$$z_{a}^{t}=z_{a}^{0},\forall a\in (A-I),\forall t\in T$$ (18)

$$c_{i}^{t}=\left\{\begin{array}{cc} c_{i}^{0}\left(1-\rho_{i}\right), & \text{if}\;\sum_{s\in S}\sum_{\tau =\text{max}\left\{1,t-d_{i}+1\right\}}^{t}x_{ist}=1,\forall i\in I,\forall t\in T\;\\ c_{i}^{0}, & \text{if}\;\sum_{s\in S}\sum_{\tau =\text{max}\left\{1,t-d_{i}+1\right\}}^{t}x_{ist}=0,\forall i\in I,\forall t\in T \end{array}\right.$$ (19)

$$z_{i}^{t}=\left\{\begin{array}{cc} z_{i}^{0}\left(1-\sigma_{i}\right), & \text{if}\;\sum_{s\in S}\sum_{\tau =\text{max}\left\{1,t-d_{i}+1\right\}}^{t}x_{ist}=1,\forall i\in I,\forall t\in T\;\\ z_{i}^{0}, & \text{if}\;\sum_{s\in S}\sum_{\tau =\text{max}\left\{1,t-d_{i}+1\right\}}^{t}x_{ist}=0,\forall i\in I,\forall t\in T \end{array}\right.$$ (20)

$$Z_{a}^{t}=z_{a}^{t}\left[1+\alpha {\left(q_{a}^{t}/c_{a}^{t}\right)}^{\beta }\right],\forall a\in A$$ (21)

$$\delta_{akw}^{t}\in \left\{0,1\right\},\;\forall a\in A,\forall w\in W,k\in K_{w}$$ (22)

The lower level is a link-based day-to-day traffic assignment model, which captures the gradual change of travelers’ behavior responding to the maintenance activities. Eqs (12)–(14) are modified formulations of the multistage Beckmann’s transformation to simulate the UE with *T* time periods [40]. The objective function (12) minimizes the total travel time for all traffic flows on the set of links in the network [40], which is subject to traffic flow conservation constraints (13) and (14) and non-negative constraint (16) of link flows. In Eq (12), $Z_{a}^{t}(v,c_{a}^{t})$ is a function of $Z_{a}^{t}$ related to traffic flows and traffic capacity on link *a*. $\sum_{a\in A}\int_{0}^{q_{a}^{t}}Z_{a}^{t}(v,c_{a}^{t})dv$ can be converted into $q_{a}^{t}∙Z_{a}^{t}(v,c_{a}^{t})$,which represents the total travel time of the network [40]. When the maintenance activity begins at time *t*, the link flow tends to evolve from the current traffic flow *q*_*a*_^*t*^ to a “target” traffic flow *q*_*a*_^*t*+1^* at a rate of *ξ*. This day-to-day evolution process is presented in Eq (15) [41]. *q*_*a*_^*t*+1^*, can be obtained from the following minimization problem:

$$\text{min}\eta {Z_{t+1}}^{'}q^{t+1*}+(1-\eta )D(q^{t},q^{t+1*})$$ (23)

Where ***q***^*t*^ is the link flow vector at the time *t*. ***Z***_*t*+1_ denotes the perceived travel time vector at the time *t*+1. ***q***^*t*+1*^ is the target link flow vector at the time *t*+1. ***Z***_*t*+1_′***q***^*t*+1*^ minimizes the total travel time in the network under a given perceived travel time ***Z***_*t*+1_. The parameter 0<*η*<1 reflects travelers’ sensitivity to travel time. *D*(***q***^*t*^, ***q***^*t*+1*^) minimizes the distance between the current link flow ***q***^*t*^ and the target link flow ***q***^*t*+1*^. *D*(***q***^*t*^, ***q***^*t*+1*^) is formulated in Eq (24). More detailed information can be seen in He and Liu [41] and He et al. [37]

$$D(q^{t},q^{t+1*})=\sum_{a\in A}\int_{q_{a}^{t}}^{q_{a}^{t+1*}}(Z_{a}^{t}(v,c_{a}^{t})-Z_{a}^{t}(q_{a}^{t},c_{a}^{t}))dv$$ (24)

Eqs (17) and (18) represent that for each link not to be maintained, its traffic capacity $c_{a}^{t}$ and free-flow time $z_{a}^{t}$ will remain constant at any time *t*, which are consistent with the initial values in the pre-maintenance period, $c_{a}^{0}$ and $z_{a}^{0}$, respectively. Eq (19) calculates the traffic capacity of each link to be maintained at the time *t*, i.e., if the maintenance activity *i* is in process at the time *t*, the traffic capacity $c_{i}^{t}$ of link *i* will drop to $c_{i}^{0}\left(1-\rho_{i}\right)$, otherwise, $c_{i}^{t}=c_{i}^{0}$. Likewise, Eq (20) formulates the free-flow time of link *i* to be maintained at the time *t*, which is $z_{i}^{t}=z_{i}^{0}\left(1-\sigma_{i}\right)$ and $z_{i}^{t}=z_{i}^{0}$ otherwise. Eq (21) is the classical BPR function proposed by U.S. Bureau of Public Roads, which is used to estimate the travel time. Eq (22) defines the type of binary variables.

## Model solution

### Simulated annealing algorithm (SA) for MMS model

It is not difficult to know that the MMS-based highway maintenance scheduling is an NP-hard combinational optimization problem [42]. SA has been proved as an effective algorithm to solve this type of problems [43]. SA is a local search algorithm based on the concept of physical annealing of solids, which can escape from being trapped into local optima by accepting worse solutions with a low possibility. This algorithm starts with an initial solution and a high initial temperature. Then, it performs the iteration following the principle as “new solution generation → objection function value calculation → acceptance or rejection of new solutions” with an annealing schedule. The solution at the end of the iteration is considered optimal. In this subsection, we propose a SA algorithm with a special initial solution representation for solving the MMS model. The framework of the SA algorithm for solving the proposed MMS model is presented in Algorithm 1.

**Algorithm 1** Pseudo code of SA procedure

//Initialization

Randomly generate a initial solution $x_{ist}^{0}$, and calculate the objection function value $f(x_{ist}^{0})$;

$x_{ist}^{best}=x_{ist}^{0}$;

*k* = 0; //The outer iteration times

*t*_*k*_ = *T*; //Temperature

**while** not stop

 //The search loop under the temperature *t*_*k*_

 **for** *i* = 1 to *L* //The inner iteration times

  Generate a new feasible solution $x_{ist}^{new}$ based on the current solution $x_{ist}^{k}$, and calculate the objection function value $f(x_{ist}^{new})$.

  **if** $f(x_{ist}^{new})<x_{ist}^{k}$

   $x_{ist}^{k}=x_{ist}^{new}$;

   **if** $f(x_{ist}^{k})<f(x_{ist}^{best})$; $x_{ist}^{best}=x_{ist}^{k}$;

   continues;

  **end if**

  Calculate the acceptance probability

  $P(t_{k})=\text{exp}[-(f(x_{ist}^{new})-f(x_{ist}^{k}))/t_{k}]$;

  **if** random(0,1)<*P*

   $x_{ist}^{k}=x_{ist}^{new}$;

  **end if**

 **end for**

 //Drop down the temperature

 *t*_*k*+1_ = drop(*t*_*k*_); *k* = *k*+1

**end** while

print $x_{ist}^{new}$

**end procedure**

#### Solution representation

Inspired by the work of Tavakkoli-Moghaddam et al. [44], the initial solution in SA algorithm is represented by a combination of two matrices *B*_|*I*|×|*S*|_ and *C*_|*I*|×|*T*|_. *b*_|*I*|×|*S*|_ ∈ *B* refers to the maintenance activity on link *i* assigned to the engineering team *s*. *c*_|*I*|×|*T*|_ ∈ *C* represents that the maintenance activity on link *i* begins at time *t*. *b*_|*I*|×|*S*|_ = 1 and *c*_|*I*|×|*T*|_ = 1 means the maintenance action for link *i* assigned to engineering team *s* starts in time *t*, which is a feasible solution of the MMS model.

#### Initial solution

**Fig 3:**
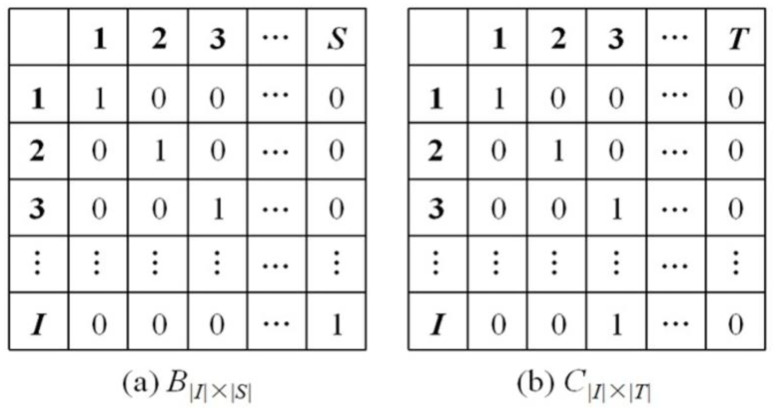
An example of the initial solution.

SA searches the globally optimal solution by generating and improving an initial solution. In this study, the initial solution is produced by constructing two matrices $B_{\left|I\right|\times \left|S\right|}^{0}$ and $C_{\left|I\right|\times \left|T\right|}^{0}$ randomly. All links to be maintained are assigned to the engineering teams following the strategy that makes the minimum variance of the total duration of the maintenance activities assigned to each engineering team. The start time of the maintenance activities is set ensuring that the number of maintenance activities start simultaneously shall not exceed the number of engineering teams. Fig 3 shows an example of the initial solution.

#### Neighborhood

We use the mutation operator to generate the neighbor solutions in this study. The mutation operator is defined as follows:

1. Mutation operator in *B*_|*I*|×|*S*|_. A maintenance activity is selected and then assigned to another engineering team.
2. Mutation operator in *C*_|*I*|×|*T*|_. A maintenance activity is selected and then started at another time.

#### Initial temperature

The initial temperature should be set as high enough to accept all new configurations. However, a high initial temperature might take more computational efforts to find the optimal solution. It is suggested by Kirkpatrick et al. [45] that the value of initial temperature should guarantee that more than 80% percent of the new solutions can be accepted. Hence, we identify the initial temperature *t*_0_ in this study using the following principle in Eq (25).

$$t_{0}=\frac{\Delta f}{\text{ln}\;p}$$ (25)

Where Δ*f* is the difference between the objective function values of two random solutions. *p* = 0.8.

#### Temperature decrement

Lower cooling speed has a higher possibility to obtain the global solution, but may consume more computation time. The following temperature shift strategy proposed by Kirkpatrick et al. [45] is applied in this work.

$$t_{k+1}=\kappa {∙t}_{k}$$ (26)

Where *κ* is the temperature decrement factor, *κ*∈[0.9,1). *k* represents the *k*th outer iteration.

#### Termination criterion

We use the freezing temperature strategy as the termination criterion for the iterative process, i.e., the annealing process terminates when the temperature drops to the freezing temperature *T*_*T*_. To ensure that the objective function value has no more significant increases, the termination temperature must be set as close to zero as possible [46].

### Augmented Lagrange algorithm for MITDS model

#### Single-level formulation of MITDS model

In general, the solution of bilevel programming is very complicated. one of the reasons is the non-convexity of bilevel programming. The non-convexity of bilevel programming indicates that even if the solution can be found, it is usually only a locally optimal solution rather than a globally optimal solution. Therefore, the exact algorithm is not applicable to the MITDS model. The key of solving bilevel programming is to find the accurate reaction function, which is difficult. In the case of continuous variables, we can solve bilevel programming using sensitivity analysis-based algorithm (SABA), which obtains the derivative relationship between variables through sensitivity analysis. However, the variable *x*_*ist*_ in the MITDS model are discrete, so the SABA cannot be used to solve the MITDS model.

A bilevel model is often solved by first transforming it to a single-level model. Inspired by this, the above bi-level MITDS model is reduced to a linear single level model by using the equivalent formulation of Eq (9).

Denote ***x***^*T*^(***e***-***x***) = 0 when 0≤***x***≤1 as the equivalent formulation of Eq (9), where ***e*** is a unit vector.


$$x^{T}\left(e-x\right)=0$$ (27)

0≤x≤1 (28)

Then, the MITDS model can be rewritten as follows:

$${\text{min}}_{q}\;M\left(q,x\right)={\text{max}}_{i\in I,s\in S}\;\{\sum_{t=1}^{T}t∙x_{ist}+d_{i}\}$$ (29)

s.t.

Eqs (2)–(8), (27) and (28).

Where ***q*** = ***q***(***x***) is defined by

$${\text{min}}_{x}\;Z_{t}\left(q,x\right)=\sum_{a\in A}\int_{0}^{q_{a}^{t}}Z_{a}^{t}(v,c_{a}^{t})dv,\forall t\in T$$ (30)

s.t.

Eqs (12)–(22).

Let Ω be the feasible region of the lower level model given fixed ***x***. The optimal-value function of the lower level model can be defined as.


$$\omega (q)\;=\underset{q_{a}^{t}\in \Omega }{\text{min}}\;Z_{t}\left(q,x\right)$$ (31)

For any feasible solution (***q***,***x***), it has ***Z***_*t*_(***q***,***x***)-*ω*(***q***)≥0. Considering that each link has a unique traffic flow pattern in the lower-level model given fixed ***x***. Hence,

$$Z_{t}\left(q,x\right)-\omega (q)=0$$ (32)

Therefore, the proposed MITDS model is be equivalent to the following single-level nonlinear programming model:

Eq (29)

s.t.

Eqs (2)–(8), (27), (28), (12)–(22) and (32).

#### Augmented Lagrange algorithm

We employ the augmented Lagrange algorithm to develop a locally convergent algorithm for the single-level MITDS model, which can incorporate the nonlinear constraint (27) into the objective function as penalty terms so as to speed up convergence.

For simplicity, we denote ***Z***_*t*_(***q***,***x***)-*ω*(***x***) as

$${\Gamma \left(q,x\right)=Z}_{t}\left(q,x\right)-\omega (x)$$ (33)

We construct the Lagrange function as

$$L\left(q,x,\gamma_{1},\gamma_{2},\varphi_{1},\varphi_{2}\right){=Z}_{t}\left(q,x\right)+\gamma_{1}\Gamma \left(q,x\right)+\gamma_{2}x^{T}\left(e-x\right)+\frac{1}{2}\varphi_{1}{\left\|\Gamma \left(q,x\right)\right\|}^{2}+\frac{1}{2}\varphi_{2}{\left\|x^{T}\left(e-x\right)\right\|}^{2}$$ (34)

Where *γ*_1_ and *γ*_2_ are two Lagrange multipliers, *γ*_1_>0, *γ*_2_>0. *φ*_1_ and *φ*_2_ are two penalty factors, *φ*_1_>0, *φ*_2_>0.

Then, we obtain an auxiliary model with the Lagrange function as the objective function as follows:

$$\underset{q,x}{\text{min}}\;L\left(q,x,\gamma_{1},\gamma_{2},\varphi_{1},\varphi_{2}\right)$$ (35)

s.t.

Eqs (2)–(8) and (13)–(22).

To solve the auxiliary model, we generate the following procedure.

*Step 1*: *Initialization*. Denote the initial values of the penalty factors as *φ*_1_^0^>0 and *φ*_2_^0^>0. Generate the initial traffic flow of each link as *q*_a_^0^>0, *a*∈*A*. Set the iteration *k* = 0 and the termination criterion *ε*>0.
*Step 2*: *Solve the lower-level model*. Solve the lower-level model Eqs (11)–(22) using the following Algorithm 2 given a fixed $x_{ist}^{k}$, *i*∈*I*, *s*∈*S*, *t*∈*T*. We obtain each link flow (*q*_*a*_^*k*^)*, *a*∈*A* and calculate the value of the optimal-value function (***x***^*k*^). Note that (*q*_*a*_^*k*^)* is not necessarily an equilibrium traffic flow pattern due to Eq (14). Then, we compute the gradient ∇*ω*(***x***^*k*^) using Eq (34).
$$\nabla \omega \left(x^{k}\right)=\left(\frac{\partial \omega \left(x^{k}\right)}{x_{1}^{k}},\cdots ,\frac{\partial \omega \left(x^{k}\right)}{x_{i}^{k}},\cdots \right),\forall k$$ (36)
$$\frac{\partial \omega \left(x^{k}\right)}{x_{i}^{k}}\;=\;\int_{0}^{q_{a}^{*}\left(x^{k}\right)}\frac{\partial Z_{t}\left(y,x\right)}{\partial x_{i}^{k}}dy,\forall k,i,a$$ (37)
  Where $q_{a}^{*}\left(x^{k}\right)$, ∀*k*,*a* is the equilibrium link flow for fixed $x_{ist}^{k}$.
*Step 3*: *Solve the submodel*. Solve the following submodel given fixed $\gamma_{1}^{k}$ and $\gamma_{2}^{k}$:
$$\underset{q,x}{\text{min}}\;L\left(q,x,\gamma_{1}^{k},\gamma_{2}^{k}\right)$$ (38)
  s.t.
  Eqs (2)–(8) and (12)–(22).
  Let (***q***^*k*+1^, ***x***^*k*+1^) be the solution.
*Step 4*: *Termination criterion test*. Denote the termination criterion *ε*>0. If $\gamma_{1}^{k}\Gamma \left(q^{k+1},x^{k+1}\right)<\varepsilon$ and $\gamma_{2}^{k}{\left(x^{T}\right)}^{k+1}(e-x^{k+1})<\varepsilon$ are both valid, the iteration stops and obtain the locally optimal solution. Otherwise, let $\gamma_{1}^{k+1}=\vartheta \gamma_{1}^{k},\;\gamma_{2}^{k+1}=\vartheta \gamma_{2}^{k}$ and *k* = *k*+1, and return to Step 2.

## Numerical experiment

### Testing highway network

**Fig 4:**
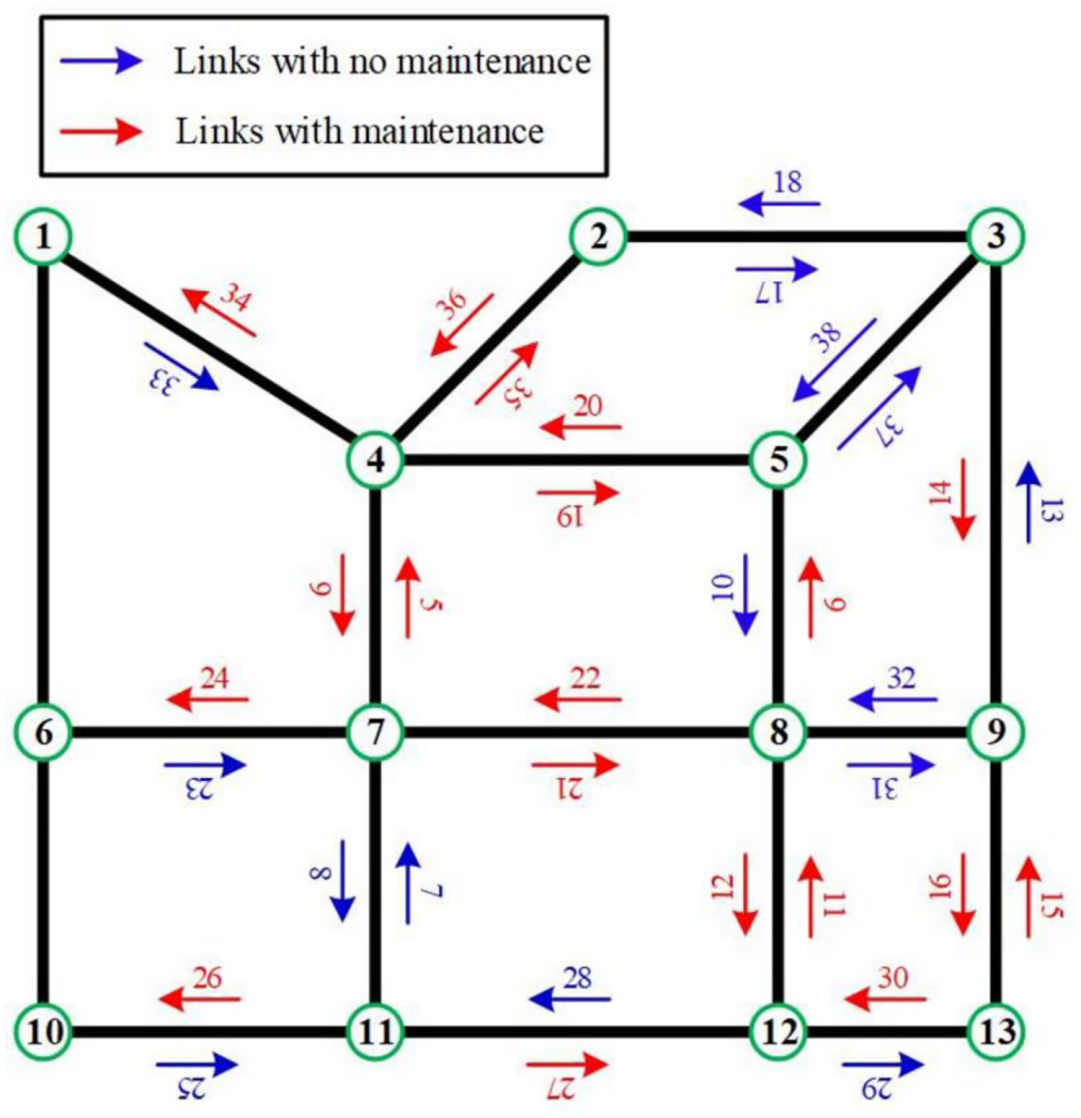
A highway network.

To verify the applicability of the proposed methodology framework, a highway network constituting 13 nodes, 38 links, 23 OD pairs and 111 paths is employed shown in Fig 4. The physical conditions (i.e., initial traffic capacity and free-flow travel time) of each link are summarized in Table 2. A maintenance project including 21 links, which are marked by red arrow lines in Fig 4, are planned to be implemented by 3 engineering teams in a finite time horizon. Table 3 shows the duration and maintenance sequence of the 21 links to be maintained. Daily traffic demands of the 23 OD pairs in the pre-maintenance period, which are assumed to remain constant during the maintenance period are reported in Table 4. The pre-maintenance traffic flow pattern on each link is assumed to be at a UE state.

**Table 2:** Physical conditions of the 38 links.

Link ID	*c*_*a*_^*0*^(veh/day)	*z*_*a*_^*0*^ (h)	Link ID	*c*_*a*_^*0*^(veh/day)	*z*_*a*_^*0*^ (h)	Link ID	*c*_*a*_^*0*^(veh/day)	*z*_*a*_^*0*^ (h)
L1	1100	0.42	L14	900	0.46	L27	1300	0.31
L2	1100	0.42	L15	1100	0.38	L28	1300	0.31
L3	1300	0.35	L16	1100	0.38	L29	1500	0.47
L4	1300	0.35	L17	1300	0.37	L30	1500	0.47
L5	1500	0.38	L18	1300	0.37	L31	1400	0.36
L6	1500	0.38	L19	1200	0.46	L32	1400	0.36
L7	1600	0.31	L20	1200	0.46	L33	1200	0.41
L8	1600	0.31	L21	1500	0.41	L34	1200	0.41
L9	1400	0.26	L22	1500	0.41	L35	900	0.47
L10	1400	0.26	L23	1400	0.43	L36	900	0.47
L11	1100	0.42	L24	1400	0.43	L37	1200	0.66
L12	1100	0.42	L25	1100	0.36	L38	1200	0.66
L13	900	0.46	L26	1100	0.36			

**Table 3:** Duration and maintenance sequence (MS) of the 21 links to be maintained.

Link ID	*d*(day)	MS	Link ID	*d*(day)	MS	Link ID	*d*(day)	MS
L2	7	1	L14	5	10	L24	6	17
L4	6	2	L15	2	7	L26	6	19
L5	4	8	L16	4	12	L27	4	18
L6	5	3	L19	6	13	L30	3	11
L9	4	5	L20	6	15	L34	7	4
L11	2	21	L21	5	16	L35	4	20
L12	2	9	L22	4	14	L36	5	6

**Table 4:** Daily traffic demands between the 23 OD pairs.

OD	*q*_*w*_(veh)	OD	*q*_*w*_(veh)	OD	*q*_*w*_(veh)	OD	*q*_*w*_(veh)
1–3	150	2–12	310	6–13	260	11–3	310
1–8	215	3–1	165	9–1	185	11–5	180
1–11	205	3–7	350	9–10	235	13–1	470
1–12	365	3–10	280	10–3	445	13–2	350
1–13	320	3–12	170	10–9	225	13–6	230
2–10	125	6–3	340	11–1	255		

The parameter values used in this experiment and algorithm are set as follows:

*ξ* = 0.9, *ρ*_*i*_ = 0.5, *σ*_*i*_ = 0.4, *α* = 0.15, *β* = 4, *η* = 0.6, *κ* = 0.95, *T*_*T*_ = 0.01, *γ*_1_ = 1, *γ*_2_ = 1, *φ*_1_ = 3, *φ*_2_ = 3, *ε* = 0.001, *ϑ* = 2, *T* = 100.

Next, Section 5.2 solves the optimal highway maintenance scheduling schemes under the proposed two maintenance strategies and compares the effectiveness of these scheduling schemes. Section 5.3 analyzes the impact of engineering teams on the increased traffic delays and makespan. Section 5.4 discusses the sensitivity of parameter *ξ*. The algorithms are implemented in Python (version 3.7.3) using the commercial solvers CPLEX (version 12.7.1). All experiments are conducted on a Windows Server 2012 R2 server with an Intel Xeon E5-2640v4 CPU (2.4GHZ) and 64GB DDR4 RAM.

### Optimal highway maintenance scheduling scheme

**Fig 5:**
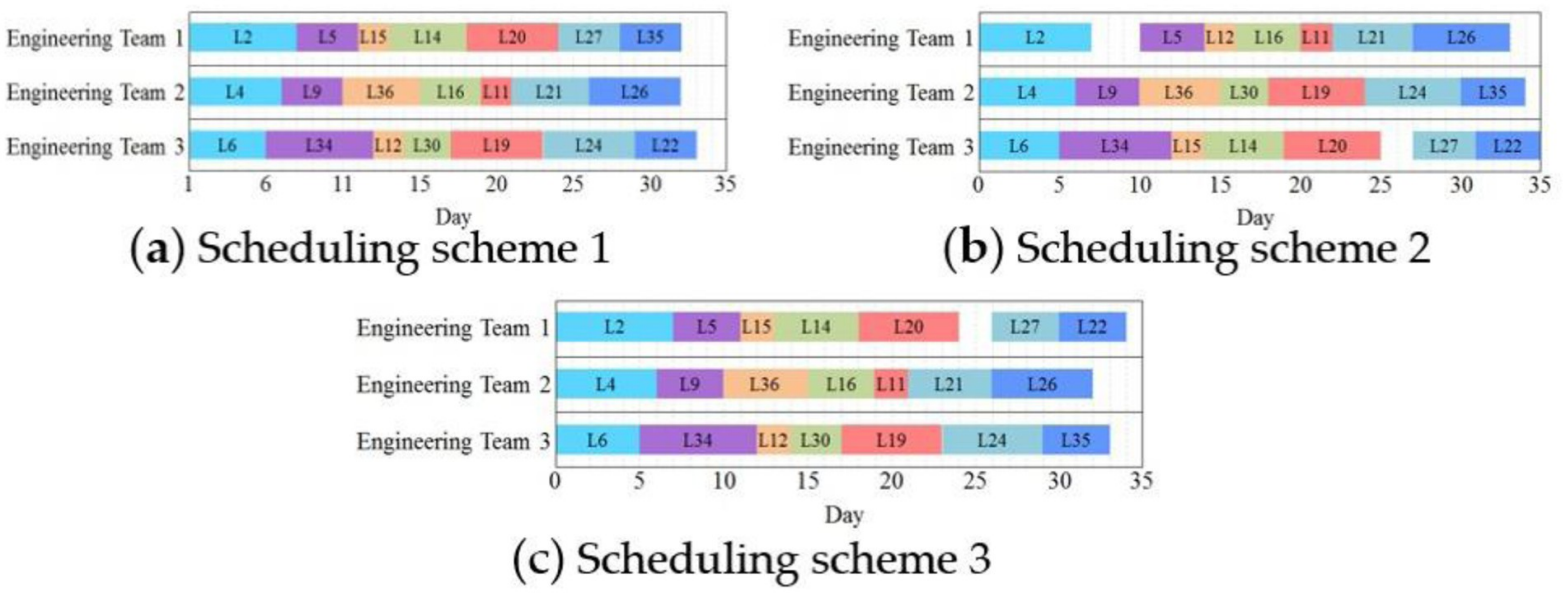
Three different highway maintenance scheduling schemes.

The optimal highway maintenance scheduling schemes generated by the proposed models are depicted in Fig 5, which shows the start time, completion time and duration of each maintenance activity and makespan as well as the maintenance sequence of each engineering team. The code on each bar in Fig 5 represents the link ID.

**Fig 6:**
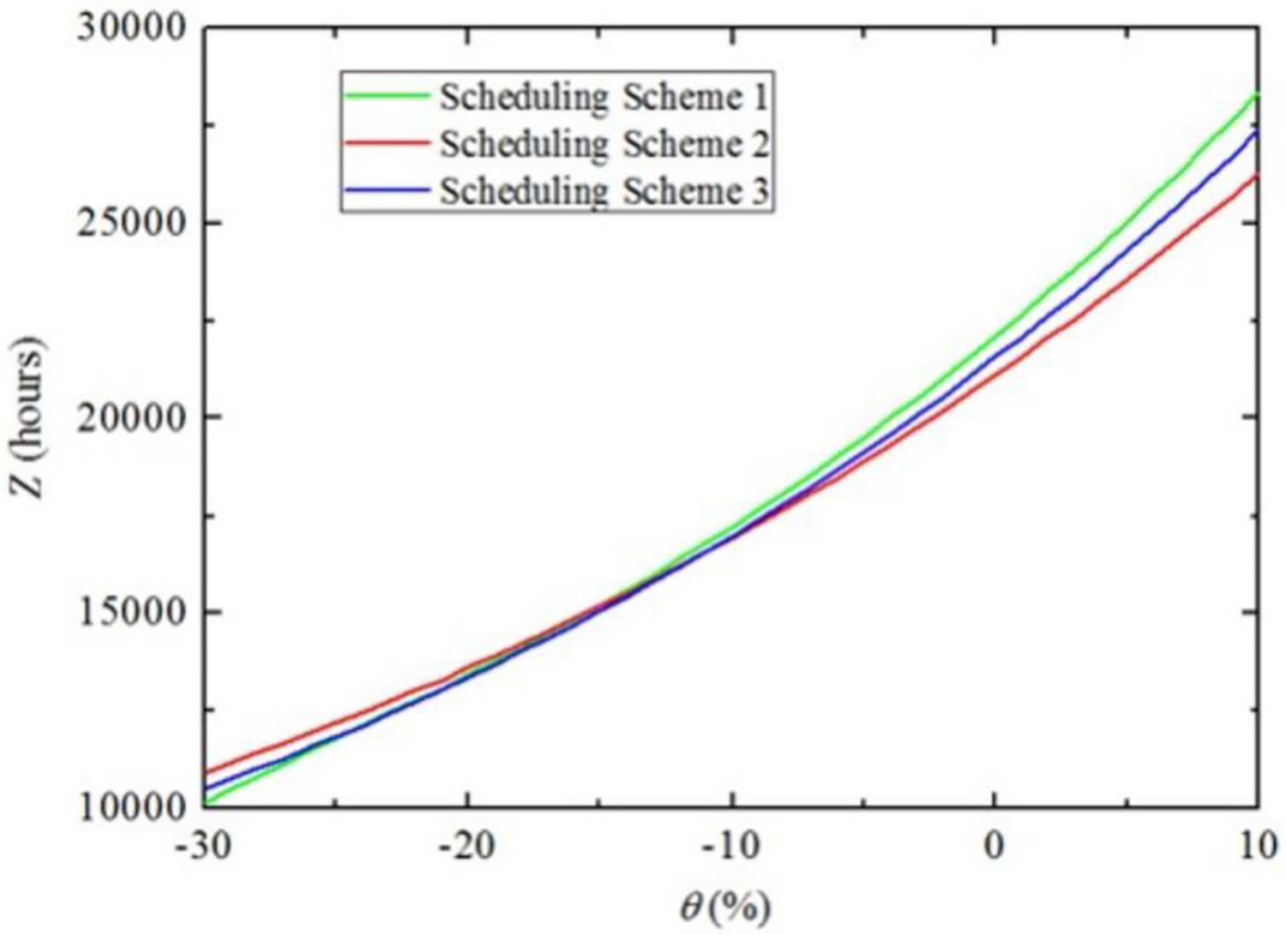
Z of the three maintenance scheduling schemes for the increase of *q*_*w*_ by *θ*.

Fig 5(a) and 5(b) indicate the optimal scheduling schemes produced by the MMS and MITDS, i.e., scheduling scheme 1 and scheduling scheme 2, respectively. Scheduling scheme 1 can guarantee minimum *M* (i.e., 33 days), but has higher *Z* (i.e., 22,098.8 hours). Compared with scheduling scheme 1, scheduling scheme 2 extends *M* by 2 days though, it reduces *Z* by 4%. Actually, MMS generates a constant optimal scheduling scheme that does not differ with the variation of *q*_*w*_. However as opposed to MMS, MITDS may produce different optimal maintenance scheduling schemes with various *q*_*w*_. Specifically, the optimal maintenance scheduling scheme under the MITDS switch to Scheduling scheme 3 shown in Fig 5(c) when *q*_*w*_ drops by 10% in this experiment shown in Fig 6, which plots *Z* of different maintenance scheduling schemes when *q*_*w*_ increases by *θ* simultaneously. Fig 6 reveals that under the objective of minimum increased traffic delays, when *θ*<-24%, scheduling scheme 1 is optimal, which means the MMS is equivalent to the MITDS, while scheduling scheme 2 and scheduling scheme 3 have outperformance when *θ*≥-10% and -24%≤*θ*<-10%, respectively.

**Fig 7:**
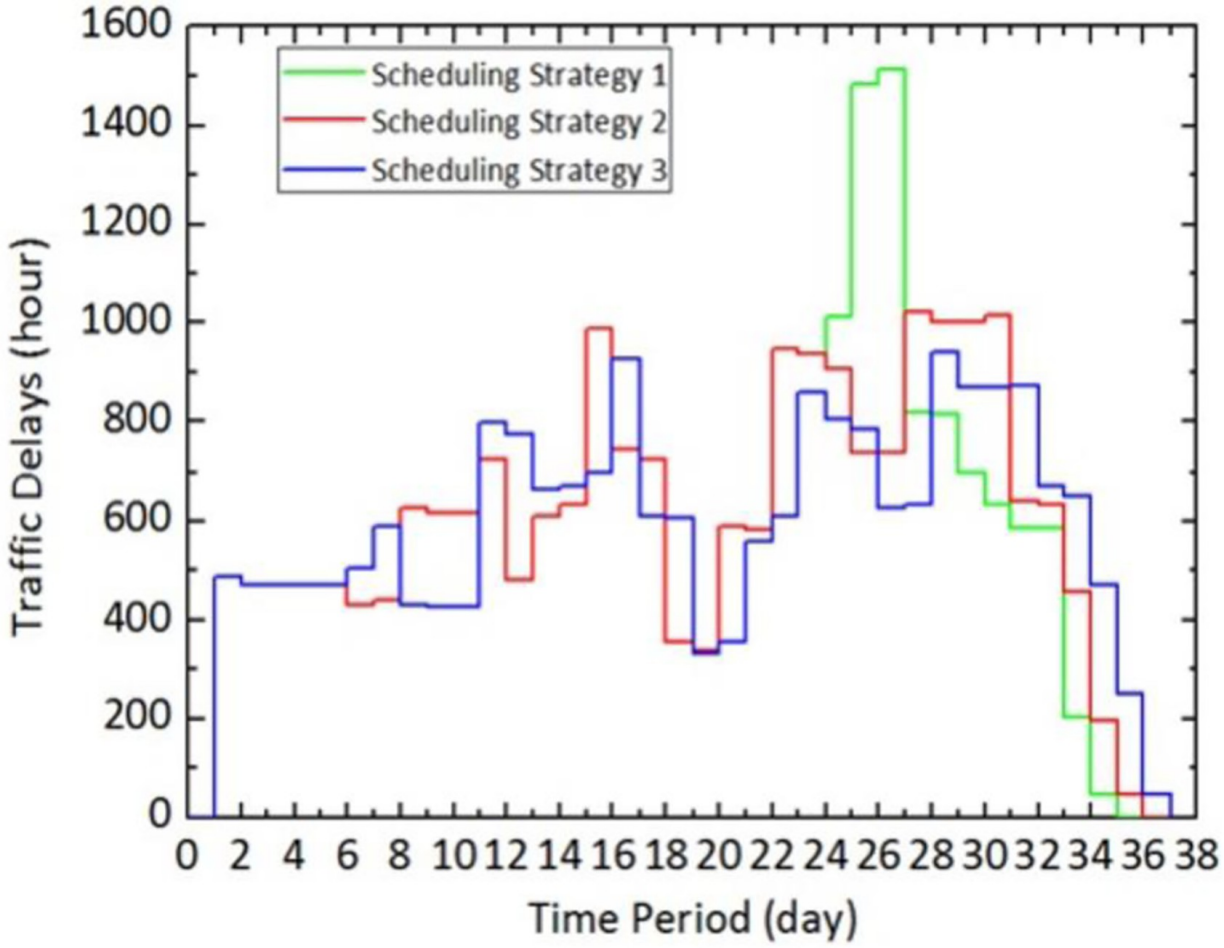
Day-to-day evolution of Z of the three different scheduling schemes (*θ* = 0).

Fig 7 demonstrates the day-to-day evaluation of *Z* for the above three different scheduling schemes when *θ* = 0. The results indicate that increased traffic delay curve of Scheduling scheme 1 has two significant peaks on days 8–10, and 24–25. The former is caused by the simultaneous maintenance activities on Link 5 and Link 9 and the latter is associated with the simultaneous maintenance activities on Link 21 and Link 27. To reduce these traffic delays, scheduling scheme 2 eliminates the two peaks by setting a small time-lag between the two maintenance activities on two parallel links.

Additionally, because of the traffic fluctuation determined by parameter *ξ*, the process of dropping the increased traffic delays to zero does not stop at the end of maintenance, but is postponed until after the end of maintenance, which can be seen in Fig 7.

### Effect of engineering teams

Since the number of engineering team is responsible for the makespan of the maintenance project and the loss of highway network capacity at the time *t*, it is necessary to identify the optimal number of engineering team. In this subsection, we analyze the optimal maintenance strategies under different numbers of engineering teams when *θ* = 0.

**Fig 8:**
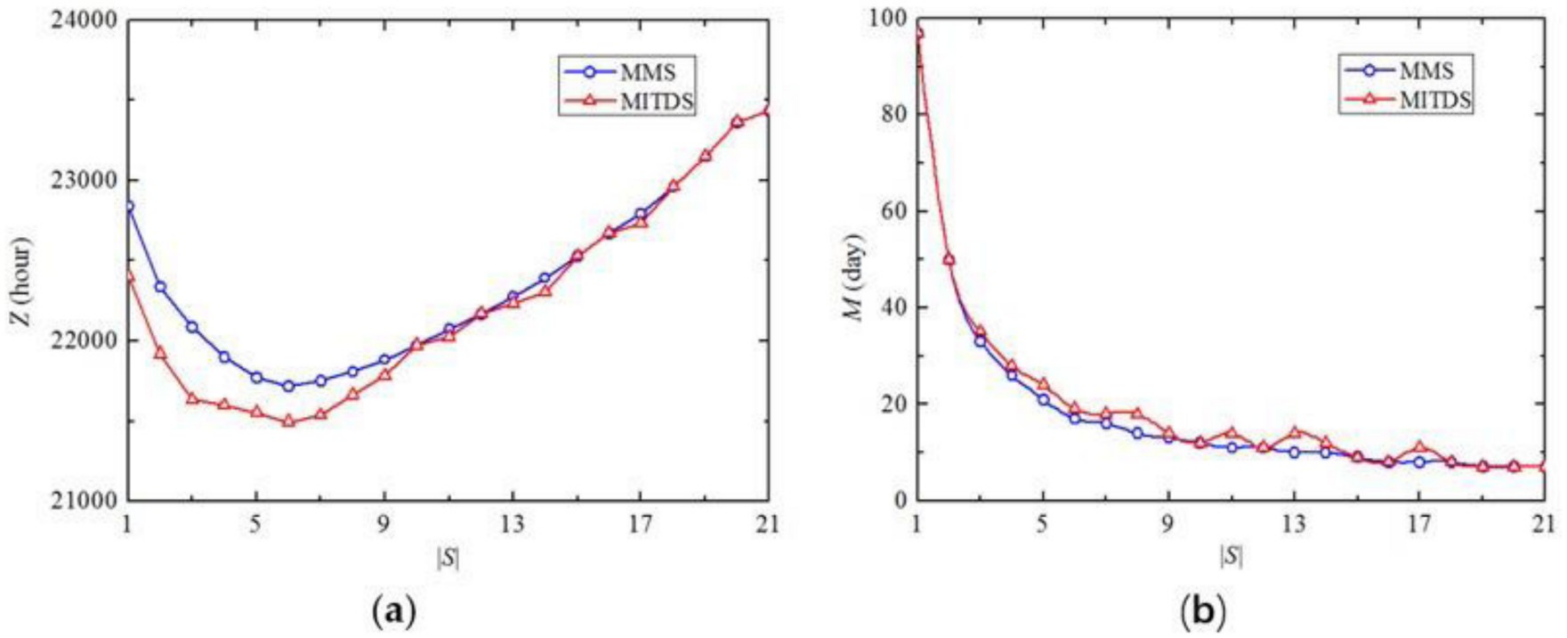
Z and M with the variation of |*S*| (*θ* = 0).

Fig 8(a) plots *Z* of the two maintenance scheduling strategies when |*S*| varies from 1 to 21. It indicates that the relationship between *Z* and |*S*| generally follows an inverted-U-shaped curve, which means that the optimal |*S*| minimizing *Z* exists, i. e., |*S*| = 6 in this experiment. *Z* decreases drastically when |*S*| varies from 1 to 6 because *M* has a steep descent from 97 days to 17 days shown in Fig 8(b). Although *M* is shortened with the increase of |*S*|, the impedance of the entire traffic network has a rapid rise due to more simultaneous maintenance activities, which explains the increase of *Z* with |*S*| changing from 6 to 21. Additionally, the marginal benefit of |*S*| tends to be lower with more |*S*|, i.e., once a certain |*S*| is exceeded, additional deployment of engineering teams has no significant reduction of *M*. Note that if the transportation agencies aim to minimize *Z*, MITDS is the preferred scheduling strategy, however, when |*S*| = 10, 12, 15, 16, 18, 19, 20 and 21, MMS can also guarantee the minimum *Z*, which is equivalent to MITDS.

### Sensitivity analysis of parameter ξ

The step-size parameter *ξ* in Eq (15) represents the weight of prediction in travelers’ perception, which indicates how drastically drivers change their paths [37]. Since the highway maintenance actions give rise to the temporal modification of the physical characteristics in the traffic network, travelers choose their new paths according to the perturbation under different inertia. Hence, in this subsection, we investigate the impact of parameter *ξ* on *Z* of the MMS and MITDS by varying the values of parameter *ξ*.

**Fig 9:**
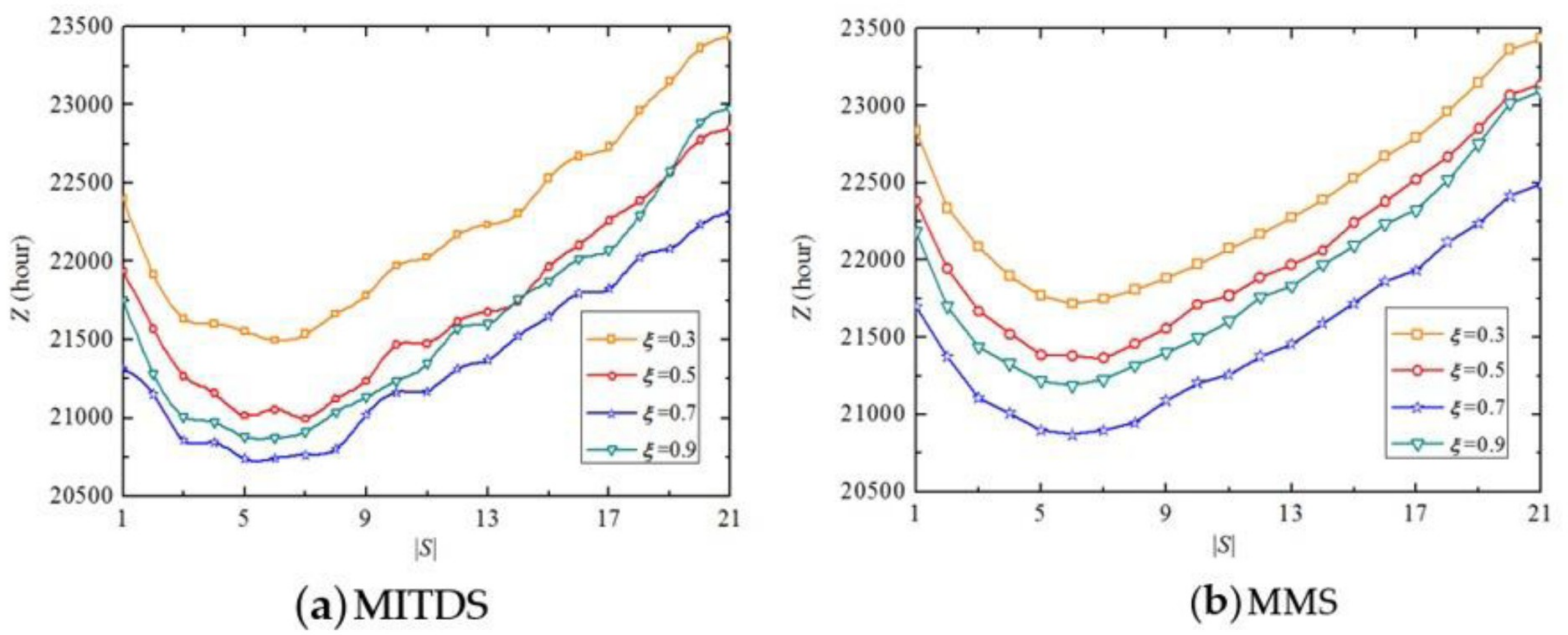
Impact of the parameter *ξ* on Z.

Let the values of parameter *ξ* vary from 0.3 to 0.9 at the interval of 0.2. The sensitivity of *Z* to parameter *ξ* is shown in Fig 9. It reveals that both the two strategies have the maximum and minimum *Z* when *ξ* = 0.3 and *ξ* = 0.7, respectively. It is found that *ξ* has no impact on |*S*|, which means regardless of the variation of *ξ*, |*S*| = 6 is always optimal for the two strategies. Note that when more than 19 engineering teams participate in the maintenance activities, the traffic delays under *ξ* = 0.9 are higher than that under *ξ* = 0.5.

**Fig 10:**
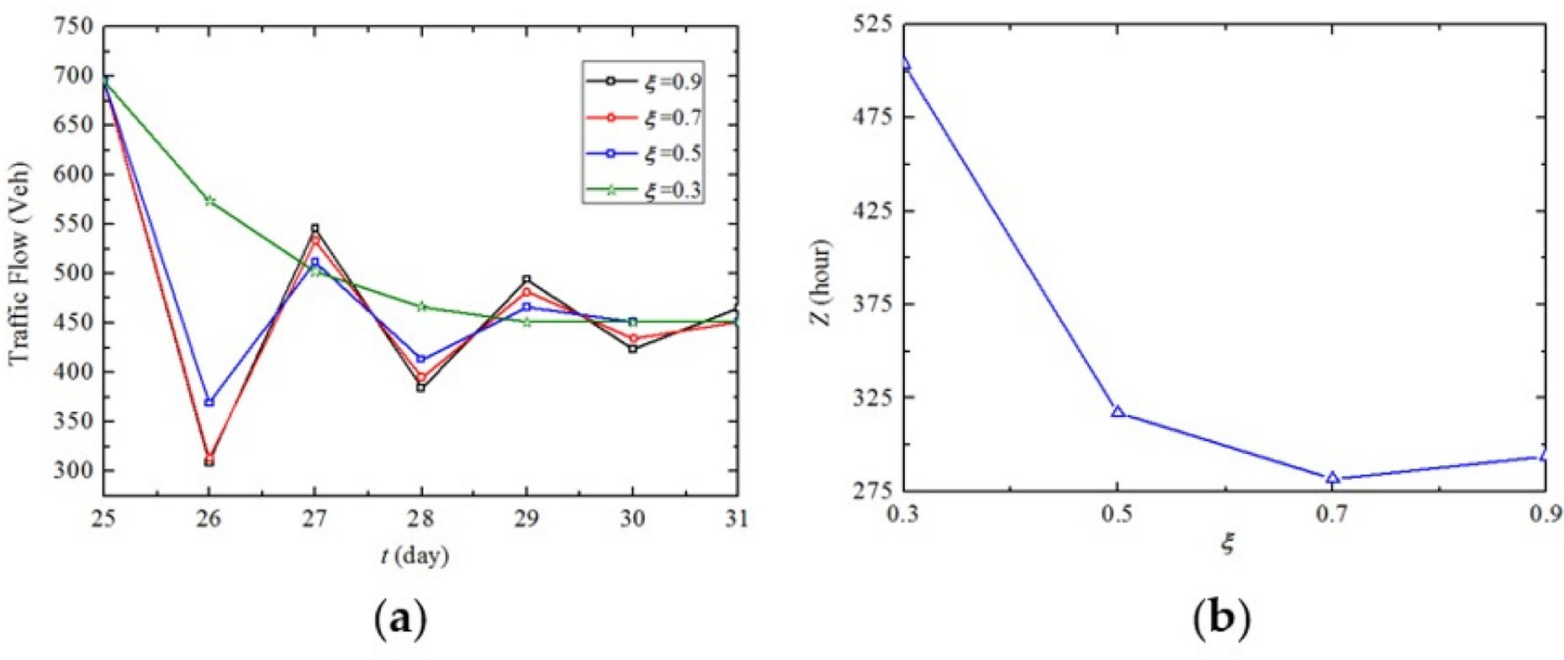
Impact of the parameter *ξ* on traffic flow and increased traffic delays on Link 24.

In order to further understand how the parameter *ξ* influences *Z*, we analyze the increased traffic delays on links under different values of *ξ*. For graph simplicity, we only show the increased traffic delays of Link 24 under MITDS with |*S*| = 3 and *θ* = 0 shown in Fig 10. Fig 10(a) indicates that the traffic flow on Link 24 fluctuates after the maintenance activity begins and reaches a new traffic equilibrium of 451 vehicles from the initial equilibrium of 695 vehicles. It is founded that the higher the value of *ξ*, the more significant the fluctuation of the day-to-day evolution of traffic flow as well as the longer time period it takes to reach the new traffic equilibrium, which complies with the previous study of He and Liu [41]. As can be seen in Fig 10(b), the increased traffic delays of Link 24 during the 6-day duration reach the minimum when *ξ* is 0.7, which verifies the results in Fig 9. Hence, it is useful for transportation agencies to provide accurate navigation information for travelers during the highway maintenance period so that traffic congestion can be mitigated.

## Conclusions

This study focuses on the highway network maintenance scheduling strategy problem, which is a follow-on problem of highway infrastructure maintenance plans. Since minimum maintenance makespan cannot ensure minimum total traffic delays, in order to illustrate the scenarios in which these two strategies are applicable, we consider and compare two maintenance scheduling strategies, i.e., MMS and MITDS. The former is formulated as a mixed integer linear programming model subject to the constraints of the quantity of manpower and the WF maintenance time sequence, which aims to ensure the minimum makespan. As for the latter, we propose a bi-level model. The upper level model determines the optimal scheduling to minimize the total increased traffic delays during the makespan. In the lower level, a modified day-to-day traffic assignment model is put forward to reflect the traffic evolution dynamics by simulating travelers’ route choice behaviors. Then, an SA algorithm with a special initial solution representation is utilized for solving the MMS model and the MITDS model is solved by an augmented Lagrange algorithm. Finally, a highway network is devised as a testbed to illustrate the effectiveness of the two proposed models. The numerical experiment reveals some important findings as follows:

1. Both MMS and MITDS can generate the optimal maintenance scheduling scheme, which guarantees not only the minimum makespan but the minimum increased traffic delays at a lower level of traffic demands in the highway network. When the traffic demands reach a certain high level, MITDS adopts the “peak-clipping” strategy to reduce the traffic delays by avoiding simultaneous maintenance activities on the parallel highway sections, which extends the makespan but has a better performance in reducing traffic delays. In the case study, when *θ*<-24%, the MMS is equivalent to the MITDS. Hence, both MMS and MITDS can work in highway networks with lower traffic volumes, while MITDS is more suitable for highway networks with higher traffic volumes. Additionally, MITDS produces various optimal maintenance scheduling schemes that differs with the variation of traffic demands, while the MMS has the unique optimal maintenance scheduling scheme.
2. Obviously under MMS, more engineering teams can shorten the maintenance makespan but has lower marginal benefit. While under MITDS, increasing the number of engineering teams may prolong the maintenance makespan instead in some cases. Under both MMS and MITDS, the relationship between the total increased traffic delays and the number of engineering teams generally follows an inverted-U-shaped curve, which means that the optimal number of engineering team minimizing the increased traffic delays and maintenance makespan exists. As the experiment result shows, both MMS and MITDS can obtain the minimum total increased travel delays when the number of engineering teams is 6.
3. Travelers’ sensitivity to congestion determines the day-to-day traffic evolution when every single maintenance activity begins and ends, which makes the traffic delays vary over time. The higher the sensitivity, the more significant the fluctuation of traffic delays, but both too high and too low travelers’ sensitivity can lead to a drastic increase of traffic delays. However, the optimal travelers’ sensitivity exists to minimize the increased traffic delays. In the case study, both MMS and MITDS have the maximum and minimum total increased travel delays when *ξ* = 0.3 and *ξ* = 0.7, respectively.

The results of the numerical experiment prove that the proposed method framework has an advantage in generating the optimal highway network maintenance scheduling schemes to reduce traffic delays and mitigate traffic congestion, which have the potential to provide reference for transportation agencies in highway asset management.

However, our work has two limitations, which should be addressed in the future research. Future work should (1) consider the uncertainties, e.g., duration of maintenance activities, traffic demands between OD pairs, etc. during the scheduling decision process; (2) consider more cases in which the splitting of maintenance activities (preemption) is allowed inclusive of integer preemption and noninteger preemption. These extensions may need the modified formulation of the scheduling model and the design of new algorithms.

## Supporting information

S1 File The data of this study. (RAR)
